# Novel elvitegravir nanoformulation for drug delivery across the blood-brain barrier to achieve HIV-1 suppression in the CNS macrophages

**DOI:** 10.1038/s41598-020-60684-1

**Published:** 2020-03-02

**Authors:** Yuqing Gong, Pallabita Chowdhury, Prashanth K. B. Nagesh, Mohammad A. Rahman, Kaining Zhi, Murali M. Yallapu, Santosh Kumar

**Affiliations:** 1 Department of Pharmaceutical Sciences, University of Tennessee Health Science Center, Memphis, TN 38163 USA; 2 Department of Microbiology and Immunology, University of Texas Rio Grande Valley, McAllen, TX 78504 USA; 3 National Institute of Environmental Health Sciences, Durham, NC 27703 USA; 4 Plough Center for Sterile Drug Delivery Solutions, University of Tennessee Health Science Center, Memphis, TN 38163 USA; 5 Laboratory of Signal Transduction, Memorial Sloan Kettering Cancer Center, New York, NY 10065 USA

**Keywords:** Drug delivery, HIV infections

## Abstract

The use of antiretroviral therapy (ART) has remarkably decreased the morbidity associated with HIV-1 infection, however, the prevalence of HIV-1-associated neurocognitive disorders (HAND) is still increasing. The blood-brain barrier (BBB) is the major impediment for penetration of antiretroviral drugs, causing therapeutics to reach only suboptimal level to the brain. Conventional antiretroviral drug regimens are not sufficient to improve the treatment outcomes of HAND. In our recent report, we have developed a poloxamer-PLGA nanoformulation loaded with elvitegravir (EVG), a commonly used antiretroviral drug. The nanoformulated EVG is capable of elevating intracellular drug uptake and simultaneously enhance viral suppression in HIV-1-infected macrophages. In this work, we identified the clinical parameters including stability, biocompatibility, protein corona, cellular internalization pathway of EVG nanoformulation for its potential clinical translation. We further assessed the ability of this EVG nanoformulation to cross the *in vitro* BBB model and suppress the HIV-1 in macrophage cells. Compared with EVG native drug, our EVG nanoformulation demonstrated an improved BBB model penetration cross the *in vitro* BBB model and an enhanced HIV-1 suppression in HIV-1-infected human monocyte-derived macrophages after crossing the BBB model without altering the BBB model integrity. Overall, this is an innovative and optimized treatment strategy that has a potential for therapeutic interventions in reducing HAND.

## Introduction

In the past two decades, the introduction of antiretroviral therapy (ART) has made significant advances in managing HIV-1/AIDS effectively^1^. However, the prevalence of HIV-1-associated neurocognitive disorders (HAND) is still a major concern, especially in the aging population^2^. The appearance and persistence of HAND are partially due to the entry of HIV-1-infected monocytes into the brain^3^. Within the CNS, HIV-1-infected monocytes differentiate into macrophages, which produce virus and spread the virus into the brain, leading to HAND^4^. HIV-1-infected macrophages serve as one of the major viral reservoirs in the CNS, provide active viral replication, even when systemic viral suppression has been achieved by ART^5^. An efficient viral suppression on CNS macrophages is important for an effective HIV-1 treatment in the brain.

The hindrance in the treatment of HAND is mainly contributed by the inability of antiretrovirals (ARVs) to cross the blood-brain barrier (BBB) after the systemic administration^6^. Previous clinical studies demonstrate that the central nervous system (CNS) Penetration-Effectiveness (CPE) of ARVs is correlated with the inhibition of HIV-1 replication in the CNS and cognitive performance in HIV-1 positive patients^7,8^. However, conventional regimens of ARVs are not sufficient to improve the penetration of ARVs and the outcomes in HAND^9^. Approximately 25% of the patients developed one or more neurological syndromes, despite receiving ART^10^. Several approaches have been employed to improve the penetration of ARVs, including ATP-binding cassette (ABC) transporters blocking method, BBB opening strategy, prodrug therapy, and nanoparticle-based drug delivery^11^. Blocking of ABC transporters may cause serious drug-drug interactions due to the abundant presence of ABC transporters in other organs. Opening the tight junctions of the BBB is not recommended because it may also allow the entry of virus and other contaminants into the brain. United States Food and Drug Administration (FDA) approval for prodrugs is complicated and difficult because prodrugs are considered as a separate chemical entity than the parent molecule^11^. Compared with other CNS delivery approaches, nanoparticle-based drug delivery approach is an attractive option for the treatment of HAND^12^.

Nanoparticle-based drug delivery system provides a relatively safe profile, protects ARVs from efflux transporters as well as from enzymatic and hydrolytic degradations, and can be used for a sustained-release of therapeutics^9^. Nanoparticles (NPs) that have been studied for brain delivery mainly include polymeric NPs such as poly(glycolic acid) (PGA), poly(lactic acid) (PLA), poly(D, L-lactide-*co*-glycolide) (PLGA), and poly(butylcyanoacrylate) (PBCA) NPs^13,14^, and metal-based NPs such as gold, silver, and zinc oxide NPs^14^. Since polymeric NPs are not only easy to be prepared and stored but highly biocompatible and biodegradable in nature, polymeric NPs have been extensively studied for delivery of therapeutics for cancer^15–18^ and HIV-1^19–22^. However, the use of polymeric NPs to deliver ARVs to the brain for the treatment of HAND is still underdeveloped.

In this study, a commonly used ARV, elvitegravir (EVG), was formulated into a poloxamer- PLGA nanoparticle (PLGA NPs). In our recent report, we have successfully encapsulated EVG into poloxamer-PLGA NPs (PLGA-EVG NPs), which improved intracellular uptake of EVG in monocyte-derived macrophages and viral suppression in HIV-1-infected primary macrophages^23^. To utilize PLGA NPs as a potential tool for drug delivery to the brain, in this work, we studied the clinically relevant properties for drug delivery applications of these PLGA NPs such as stability and the safety profile. We also identified the interaction of human serum (HS) proteins and PLGA NPs, and the safety profile of the HS-PLGA NP complex that includes their hemo- and cyto-compatibility. Further, we performed the transmigration study of PLGA NPs in the *in vitro* BBB model, and subsequently assessed the efficacy of PLGA-EVG NPs on viral suppression of HIV-1-infected primary macrophages after crossing the BBB model. Overall, PLGA NPs provided a promising delivery of EVG across the BBB model and has a potential for therapeutic interventions in reducing HAND.

## Results

### Formulation design and *in vitro* release of PLGA-EVG NPs

**Figure 1:**
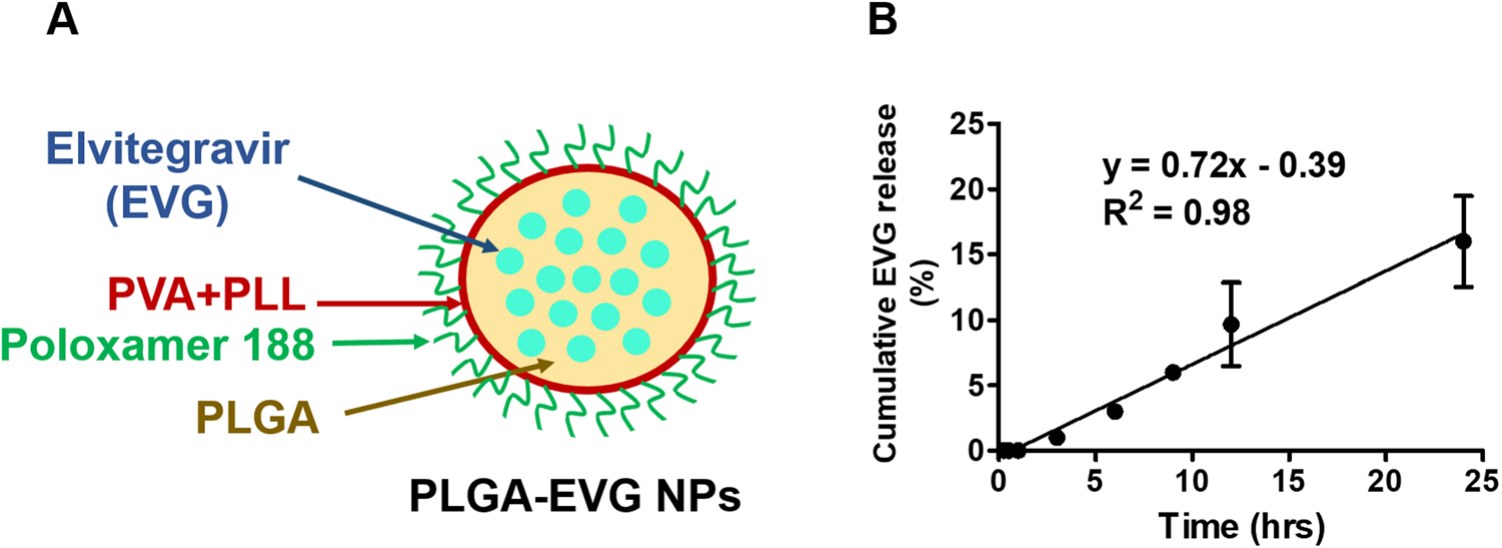
Formulation design and *in vitro* release of PLGA-EVG NPs. (**A)** A hypothetical structural representation of PLGA-EVG NP **(B)** *In vitro* release profile of PLGA-EVG NPs. Mean ± SEM values were graphed from 3 independent experiments.

The PLGA-EVG NP formulation in our study is an oil-in-water (o/w) emulsion. A hypothetical structure of PLGA-EVG NP is described in Fig. 1A. PLGA was the main polymer core to hold EVG molecules, PVA was used to stabilize the emulsion; poloxamer 188 was used as a stabilizer and to provide a better brain penetration; PLL was used to provide slightly positive charge, which helps internalize into the cells. The *in vitro* release of PLGA-EVG NPs in cell culture media is shown in Fig. 1B. The release of EVG from PLGA NPs followed a zero-order release profile with r^2^ of 0.98 within 24 h. Only ~15% of cumulative EVG release was observed at 24 h, which suggested the potential controlled release profile of PLGA-EVG NPs. The drug release from PLGA NPs may be related to the drug/PLGA loading ratio, NP protected layer stability, and physicochemical properties of EVG.

### Size and zeta potential of PLGA NPs

The size of freshly made PLGA NPs was approximately 135.7 ± 1.5 nm in PBS (Table 1). No specific change in particle size and zeta potential of NPs was observed for 7 days when stored at room temperature. PLGA NPs can be stored at 4 °C and −20 °C for at least 30 days with no change on size and zeta potential. PLGA NPs showed a stable particle size in 1 mM HEPES buffer within the pH range of 1–11 (Table 2). PLGA NPs showed a positive charge (~7 mV) at all the recorded pH values except pH 4, 5, and 11. A higher positive charge (~20 mV) of PLGA NPs was observed at the pH range of 4–5, and a lower charge (~3 mV) of PLGA NPs was observed at pH 11.

**Table 1:** Size and zeta potential of PLGA NPs stored at room temperature, 4 °C, and −20 °C.

Days	Room temperature								4 °C		−20 °C	
	Day 0	Day 1	Day 2	Day 3	Day 4	Day 5	Day 6	Day 7	Day 7	Day 30	Day 7	Day 30
Size (nm)	135.7 ± 1.5	134.4 ± 0.7	135.5 ± 0.5	135.7 ± 0.4	132.6 ± 0.6	134.2 ± 1.3	135.9 ± 1.4	134.6 ± 1.2	132.1 ± 0.5	133.1 ± 0.5	131.9 ± 0.2	132.9 ± 0.3
Zeta potential (mV)	0.11 ± 0.5	−0.53 ± 0.3	−0.18 ± 0.3	−0.02 ± 0.18	0.62 ± 0.2	−0.37 ± 0.2	0.00 ± 0.2	−0.18 ± 0.3	−0.25 ± 0.2	−0.22 ± 0.3	−0.02 ± 0.3	−0.12 ± 0.3

Mean ± SEM values were calculated from three measurements. Size and zeta potential of NPs from different storage conditions were compared with day 0, room temperature sample.

**Table 2:** Size and zeta potential of PLGA NPs in 1 mM HEPES buffer from pH 1 to pH 11.

	pH 1	pH 3	pH 4	pH 5	pH 6	pH 7.4	pH 8	pH 10	pH 11
Size (nm)	132.2 ± 1.0	132.5 ± 0.8	134.4 ± 0.8	133.8 ± 0.9	132.1 ± 0.4	132.0 ± 0.4	131.5 ± 1.2	126.9 ± 0.7	128.3 ± 1.5
Zeta potential (mV)	7.02 ± 0.2	6.36 ± 0.6	18.87 ± 1.8	22.27 ± 0.4	7.72 ± 1.3	7.94 ± 0.75	6.90 ± 0.55	5.87 ± 0.34	3.40 ± 0.31

Mean ± SEM values were calculated from three measurements.

### Evaluation of human serum protein binding on PLGA NPs

**Figure 2:**
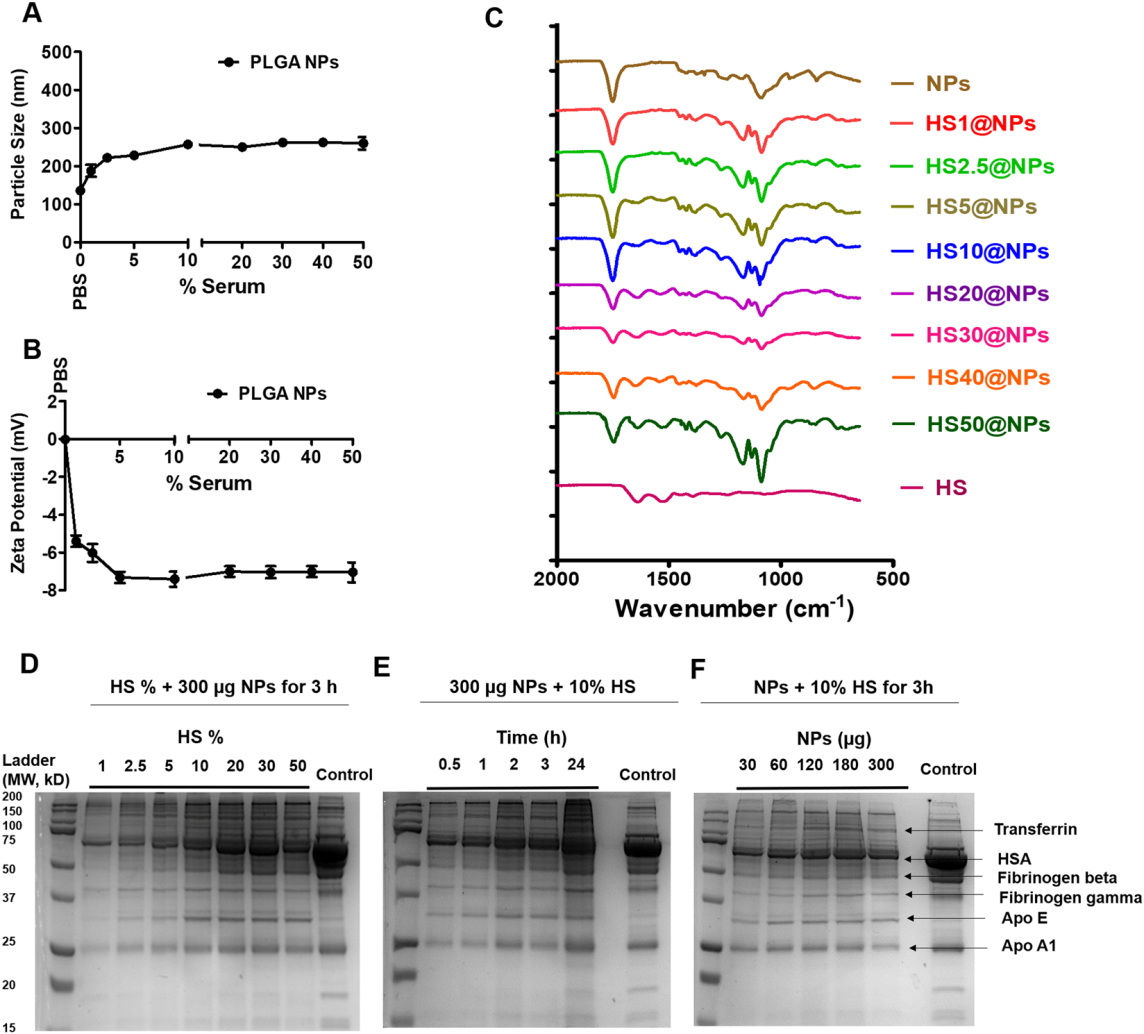
Evaluation of human serum protein binding on PLGA NPs. (**A**) Particle size of PLGA NPs in 1–50% human serum. **(B)** Zeta potential of PLGA NPs in 1–50% human serum. **(C)** FTIR spectra of human serum, PLGA NPs and HS@NPs. **(D,E)** SDS-PAGE of adsorbed human serum proteins on PLGA NPs. Data represents human serum proteins bind to PLGA NPs depending on the **(D)** concentration of human serum, **(E)** incubation time, and **(F)** concentration of PLGA NPs. Mean ± SEM values are from 3 independent experiments.

It is expected that HS protein binding occurs when NPs are taken by human body^24^. Upon introduction of NPs into the body, NPs dynamically interact with all the serum proteins and form the NP-protein complex^25^. Understanding the interaction of NPs with HS can predict their safety and efficacy profiles when used in the clinic^24^. Therefore, a DLS assay of PLGA NPs was investigated in PBS and 1–50% HS. A slight increase in particle size was observed for PLGA NPs in 1–50% HS (Fig. 2A). HS proteins are loosely bound to the PLGA NPs, leading to this slight increase in particle size. Zeta potential of PLGA NPs was observed to be negative in 1–50% HS (Fig. 2B). This negative charge is expected, because HS is negatively charged in pH 7.4 PBS. The negative zeta potential indicates that PLGA NPs are stable and safe for therapeutic applications. PLGA NPs did not precipitate in the presence of HS. To confirm the human serum binding on PLGA NPs, we performed a FTIR analysis of PLGA NPs, HS, and HS@NPs. The FTIR data was presented from 600 to 1800 cm^−1^ to observe the HS protein peaks. The peaks at 1661 cm^−1^ and 1541 cm^−1^ was found in HS and HS@NPs due to the amide bond stretch of the HS protein. The peak extent of the HS proteins in HS@NPs showed a concentration dependent increase at increasing percentage of HS (Fig. 2C). Moreover, we further confirmed the PLGA NPs-protein interaction using SDS-PAGE analysis, in which, it can be observed the HS proteins binding on HS@NPs. The identified major proteins that bind on NPs are: transferrin, human serum albumin (HSA), fibrinogen beta, fibrinogen gamma, apolipoprotein E (Apo E), and apolipoprotein A1 (Apo A1) according to the molecular weight^26^. We found that HS proteins were adsorbed to the surface of PLGA NPs when the concentration of HS increased (Fig. 2D). With an increased incubation time, an increased protein binding on the PLGA NPs was observed (Fig. 2E). HS protein binding also showed an increased pattern when the amount of PLGA NPs was increased up to 180 µg while HS protein binding decreased when incubated with more than 180 µg of NPs (Fig. 2F). This indicates the saturation of binding with NPs due to the limited HS amount and incubation time.

### Hemocompatibility of PLGA NPs

**Figure 3:**
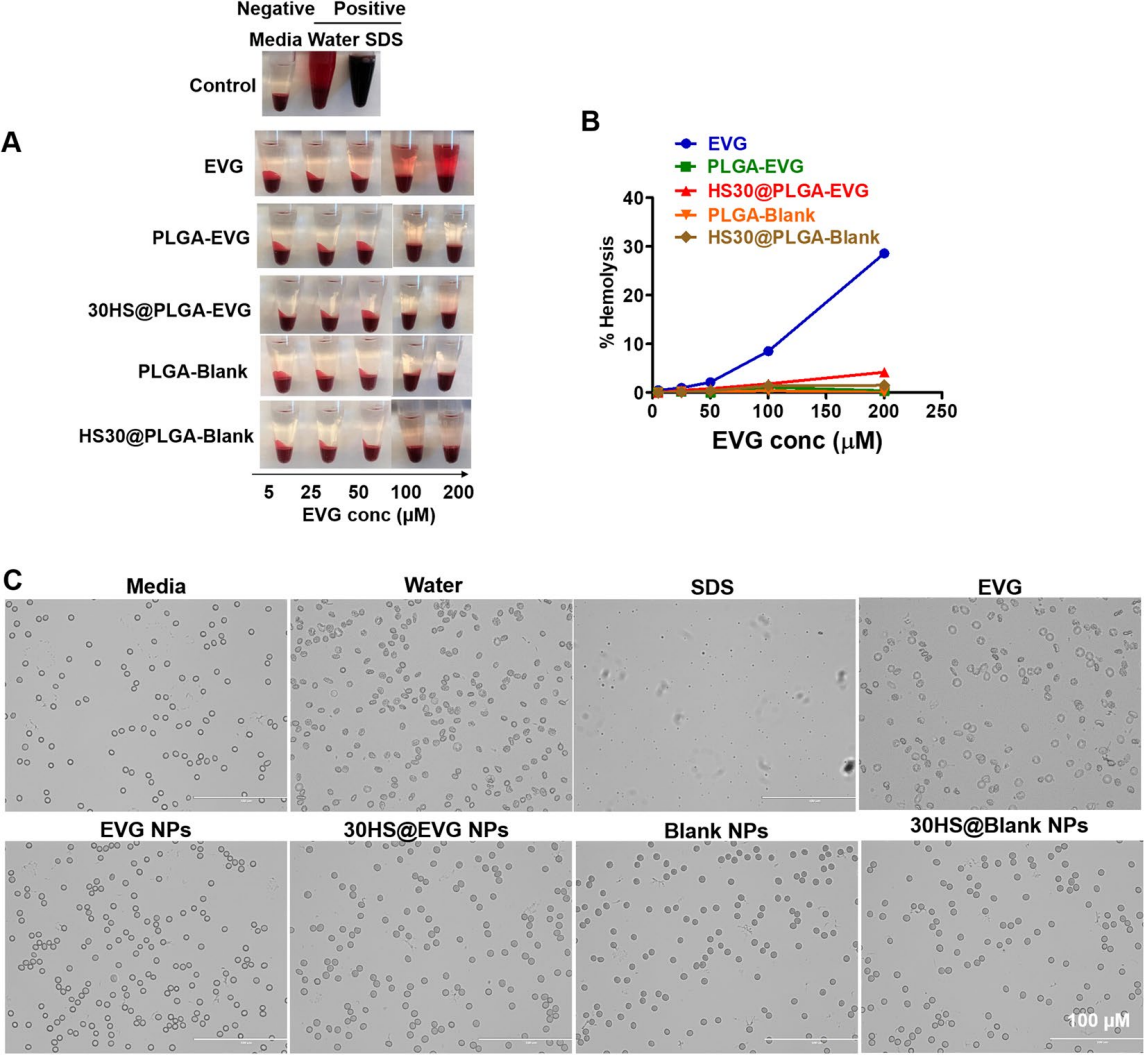
Hemocompatibility of PLGA NPs. (**A)** Visual inspection of the tubes containing RBCs after exposure to 5–200 µM EVG, EVG NPs, HS30@EVG NPs, Blank NPs, and HS30@Blank NPs. **(B)** Hemolysis of RBCs upon incubation with 5–200 µM EVG, EVG NPs, HS30@EVG NPs, Blank NPs, and HS30@Blank NPs. **(C)** Microscopic images of RBCs treated with 200 µM EVG, EVG NPs, HS30@EVG NPs, Blank NPs, and HS30@Blank NPs. 1X PBS was used as negative control, water was used as positive control #1, and sodium dodecyl sulfate (SDS) was used as positive control #2. % of hemolysis was calculated based on the positive control #1. Data represents triplicates.

Hemocompatibility tests evaluate the effects on RBCs when introduce a foreign compound to the blood. Both visual inspection (Fig. 3A) and spectrophotometric measurement of hemoglobin release (Fig. 3B) suggested that PLGA NPs do not cause hemolysis even at the highest concentration (200 µM) that was used in the assay. A dose dependent hemolysis was observed in RBCs treated with EVG native drug. At the highest tested concentration (200 µM), EVG native drug caused ~30% hemolysis while PLGA-EVG NPs did not cause hemolysis. Similarly, HS30@EVG NPs also caused low hemolysis (~4%) compared to EVG native drug (~30%). Moreover, EVG native drug caused morphology change on RBCs, while PLGA-EVG NPs and HS30@EVG NPs caused no change on the RBC morphology (Fig. 3C). The hemocompatibility of PLGA NPs could be because of the biocompatibility of the polymer and the HS adsorption on the PLGA NPs.

### Toxicity evaluation of PLGA-EVG NPs in monocytes-derived macrophages (MDM)

**Figure 4:**
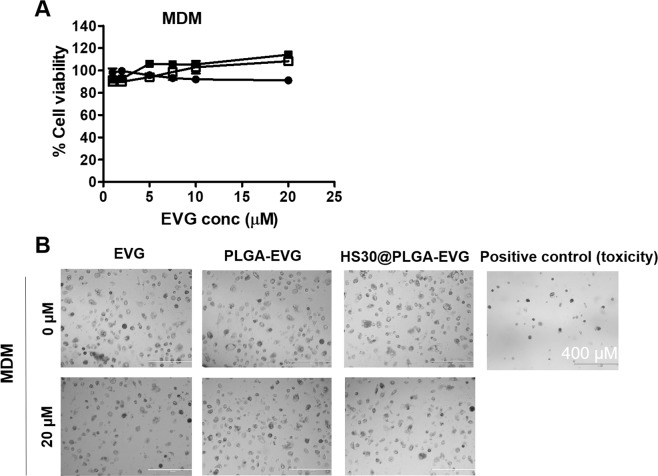
Toxicity evaluation of PLGA-EVG NPs in MDM. (**A)** Cell viability of MDM with the treatment of 0–20 µM EVG, PLGA-EVG NPs, and 30% human serum-PLGA-EVG NPs complex (HS30@PLGA-EVG) from XTT assay. **(C)** Microscopic images of MDM with the treatment of 0 and 20 µM EVG, PLGA-EVG NPs, and HS30@PLGA-EVG. 5% acetonitrile was used as a positive control. Mean ± SEM values are from 5 replicates.

It is important to carefully control the biocompatibility and cytotoxicity of a biomaterial for the potential use in the clinic^27^. Thus, we evaluated the toxicity profile of PLGA-EVG NPs with monocytes-derived macrophages (MDM). Over the range of 0–20 µM EVG, PLGA-EVG or 30HS@EVG NPs, ~100% cell viability was observed with all these groups when compared to control cells (Fig. 4A). No change in cell morphology was observed even after treating with the highest concentration (20 µM) of EVG free drug/PLGA-EVG NPs/HS30@PLGA-EVG (Fig. 4B).

### Internalization mechanism of the PLGA NPs in MDM

**Figure 5:**
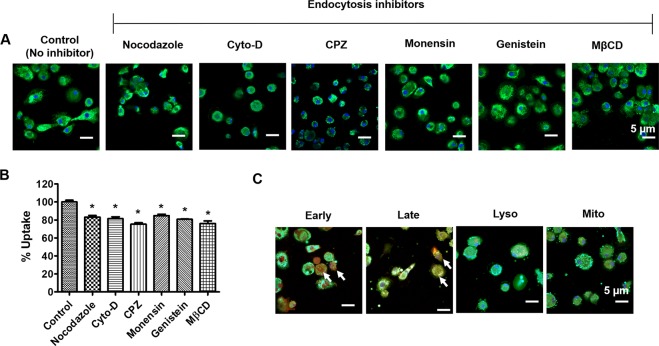
Internalization mechanism of the PLGA-C6 NPs in MDM. (**A)** Cellular uptake of PLGA-C6 NPs. in the presence of various endocytosis inhibitors. Bar equals to 5 µm in confocal images. **(B)** Relative cellular uptake percentage calculated from the mean fluorescence intensity measured by flow cytometry in MDM. Mean ± SEM are from 3 measurements. *p < 0.05. **(C)** Co-localization evaluation of PLGA-C6 NPs (green) in presence of markers (red) for early endosome, late endosome, lysosome, and mitochondria. DAPI was used to stain nuclei (blue). White arrow indicates the co-localization of PLGA-C6 NPs with early- and late- endosome markers in the cells. Cells were visualized under 400× magnification (Bar = 5 µm).

In our recent report, we showed a time- and concentration-dependent uptakes of PLGA NPs in monocytes^23^. To further study the endocytosis pathways of these PLGA NPs in macrophage, various endocytosis inhibitors were utilized. The inhibitors include nocodazole, Cyto-D, CPZ, monensin, genistein, and MβCD, which suppress microtubule-related internalization, macropinocytosis, clathrin-mediated endocytosis, lysosome-involved internalization, caveolae-mediated pathways, and caveolae-/clathrin-mediated endocytosis, respectively^28,29^. To detect by flow cytometry, a fluorescence probe, C6 (green) was encapsulated in PLGA NPs. The inhibition effect of all the endocytosis inhibitors on the internalization of PLGA-C6 NPs in MDM are minor, indicating that the internalization of PLGA NPs in MDM may relate to a combination of endocytosis pathways (Fig. 5A,B). We further examined the sub-cellular fate of PLGA NPs using a confocal microscopic analysis (Fig. 5C). PLGA NPs were efficiently internalized in MDM after 2.5 h exposure. A co-localization of PLGA-C6 and early endosome marker were observed, but less co-localization was observed in the presence of late-endosomal marker, and no co-localization was observed in the presence of the lysosomal and mitochondria marker. This suggests that PLGA NPs can escape from endo-lysosomal compartments and deliver the therapeutics to the macrophages efficiently.

### *In vitro* BBB model and TEER assessments

**Figure 6:**
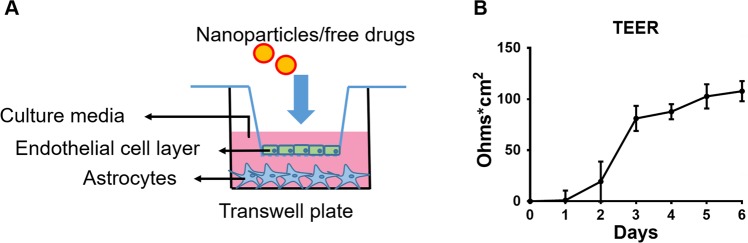
*In vitro* BBB model. (**A**) Graphical representation of the *in vitro* BBB model. **(B)** TEER measurements were obtained by applying a transendothelial current to the membrane and then testing the resistance (current, Ohm) multiplied by the area (cm^2^) of the endothelial monolayer (Ohm⋅cm^2^) by using EVOM2 meter. (C). Mean ± SEM values were graphed from 3 independent experiments.

The *in vitro* BBB model used in this study was co-cultured endothelial cells (bEnd.3) with astrocytes (C8-D1A) in a Transwell^®^ plate as described in the method section (Fig. 6A). The bEnd.3 cell are a commercially available cell line and a useful BBB-mimicking system for biological and pharmacological research because it expresses efflux transporters P-glycoprotein (P-gp) and breast cancer resistance protein (BCRP)^30,31^. A bEnd.3/C8-D1A co-culture model has shown to present significantly higher tight junctions than the monoculture of bEnd.3^32^. In our study, TEER values were measured for 6 days after co-culturing. We observed that the TEER value reached a plateau on day-4 and it was stable from 4 to 6 days **(**Fig. 6B). A mean TEER value of 100 to 120 Ohms × cm^2^ was observed in the confluent BBB model, which is consistent with the literature report^32^. All transmigration experiments were conducted after membrane integrity was stable with consistent TEER values.

### Transmigration of PLGA-C6 NPs across the *in vitro* BBB model

**Figure 7:**
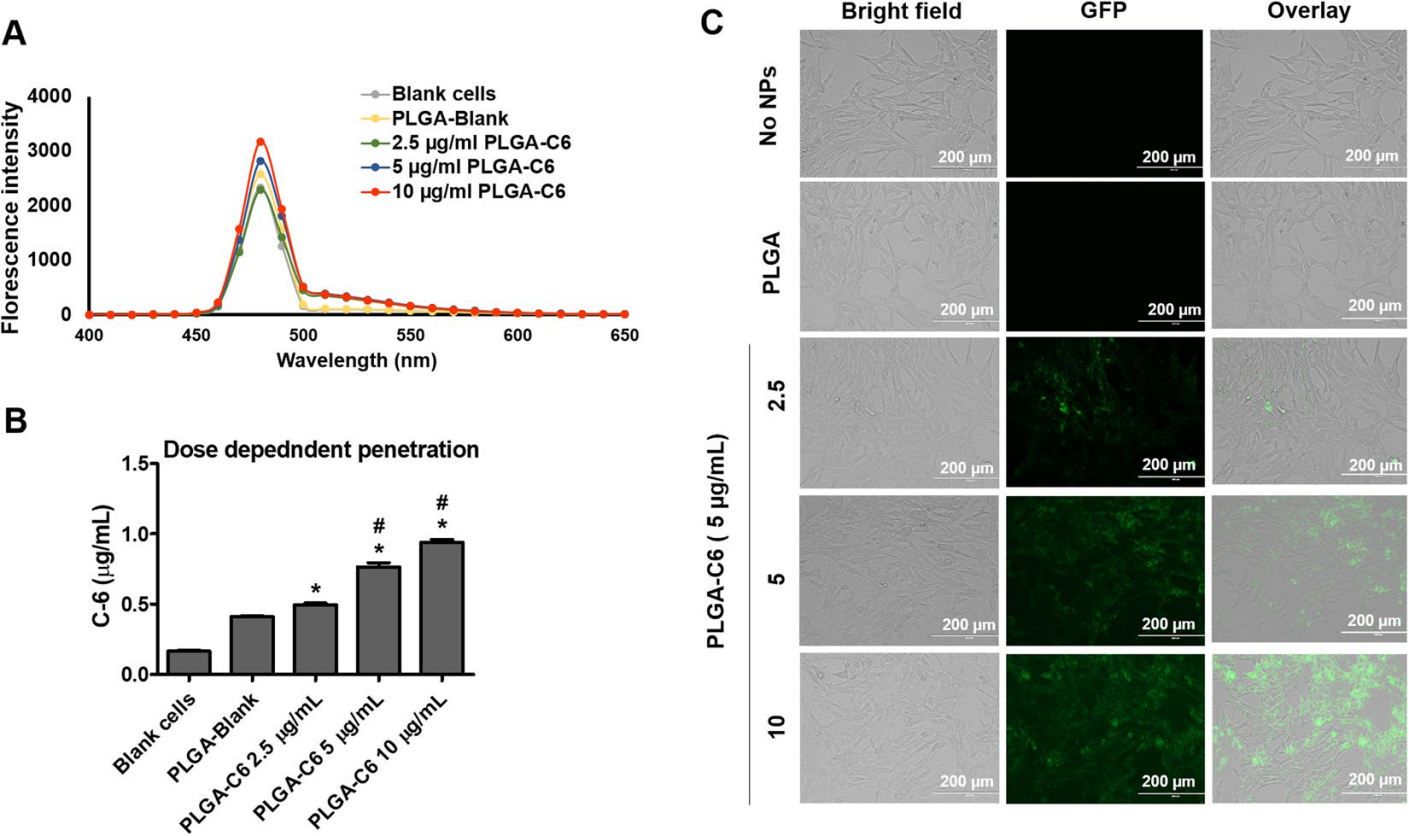
Dose dependent penetration of PLGA NPs across the BBB *in vitro*. (**A**) Quantitative penetration of PLGA-C6 after 24 hours exposure of PLGA-C6 to the top insert. The florescence intensities of the media from bottom chambers was determined by SpectraMax M2e UV spectrometer. Fluorescence was studied by exciting at 488 nm and emission between 400–650 nm. (**B)** C6 concentration in the media was calculated using a C6 calibration curve with the matrix of media. C6 concentration of 1 µg/mL represents 302101 mean florescence intensity (area under the curve). **(C)** Bright field, fluorescent, and overlay images of astrocytes from bottom chambers after 24 hours exposure of PLGA-C6 to the top insert. PLGA-C6 was taken up dose-dependently by the astrocytes in bottom chambers after crossing the BBB. The green florescence was visualized using an EVOS® FL Imaging System. Scale bar = 200 µm. Mean ± SEM values were graphed from 3 measurements. *Indicate p < 0.05 compared with PLGA-Blank, ^#^Indicate p < 0.05 compared with the previous concentration.

The characterization of the PLGA-C6 NPs was reported previously, which demonstrated a time- and dose-dependent uptakes in monocytes^23^. To test the transmigration of PLGA-C6 NPs across the *in vitro* BBB model, three different concentrations of PLGA-C6 NPs were exposed in the upper chamber, and the culture media in the bottom chamber was collected after 24 hours. The penetration of PLGA-C6 NPs was examined by quantifying the fluorescence intensity of the culture media, which was collected from the bottom chamber. We observed a dose-dependent increase in fluorescence intensity from media samples, which suggests a dose-dependent penetration of PLGA-C6 NPs across the BBB model (Fig. 7A). This finding is also supported by a quantification of C6 from the same media. C6 concentrations of the media collected from the bottom chamber were calculated using a C6 calibration curve. The penetration of C6 across the BBB model also followed a dose-dependent profile, and all the PLGA-C6 groups showed significant increase in C6 fluorescence intensity compared with PLGA-Blank after crossing the BBB model (Fig. 7B, *p < 0.05 compared with PLGA-Blank, #p < 0.05 compared with the previous concentration). These results were also validated by the qualitative imaging of recipient astrocytes, which showed that PLGA-C6 NPs were taken up by cells in a dose-dependent manner after crossing the BBB model. The highest fluorescence was observed in cells treated with the highest concentration of PLGA-C6 NPs **(**Fig. 7C**)**.

### Increased penetration of PLGA-EVG NPs in the *in vitro* BBB model

**Figure 8:**
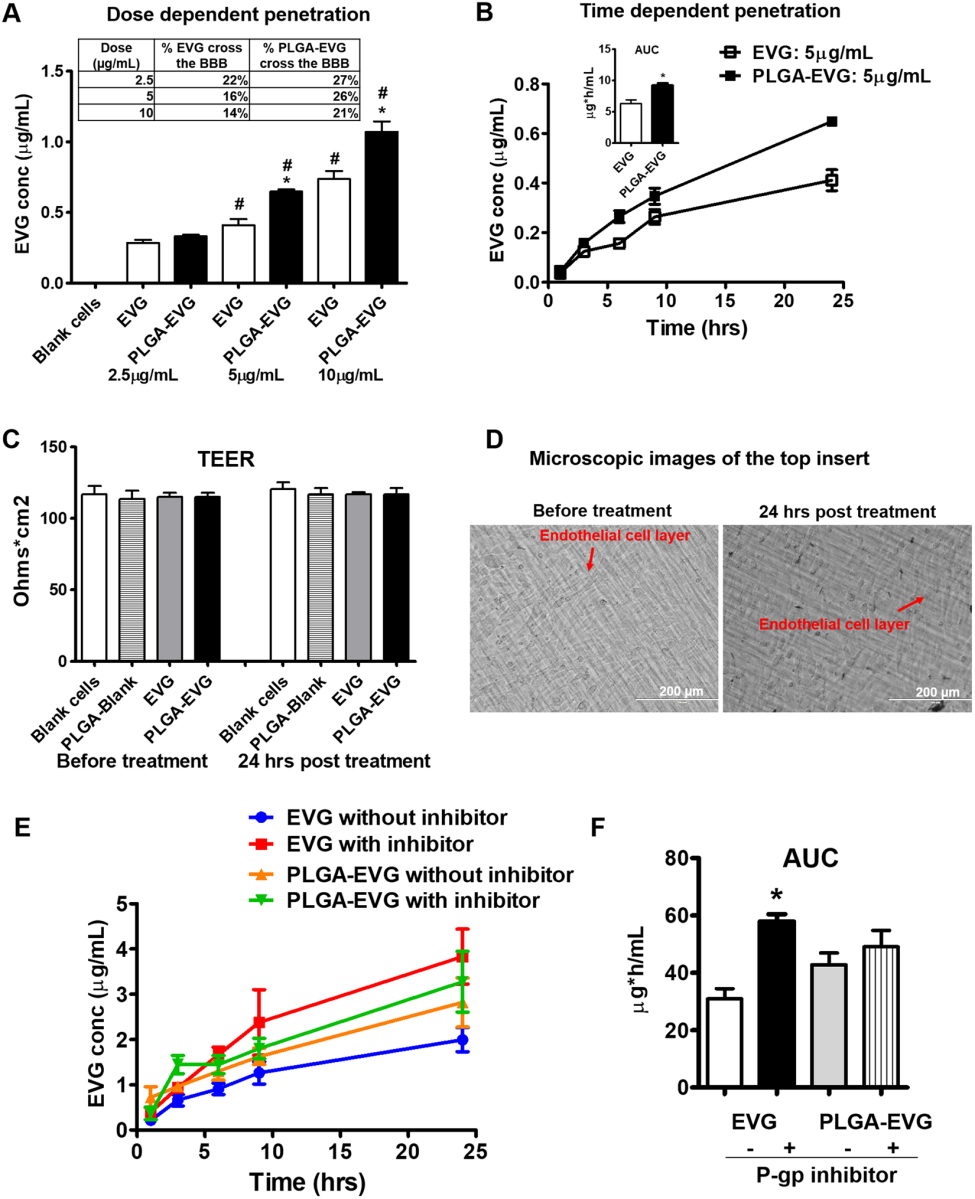
Dose-/time-dependent penetration of EVG and PLGA-EVG NPs across the BBB *in vitro*. (**A)** Dose dependent penetration profile of EVG/PLGA-EVG NPs across the BBB *in vitro*. Mean ± SEM values were graphed from 3 independent experiments. *Indicate p < 0.05 compared with native EVG drug, # indicate p < 0.05 compared with the previous EVG/PLGA-EVG concentrations. **(B)** Time dependent penetration profile of EVG/PLGA-EVG NPs across the *in vitro* BBB. Quantitative penetration of EVG was determined by LC-MS/MS. Mean ± SEM values were graphed from 3 independent experiments. *Indicate p < 0.05 compared with native EVG drug, **(C)** Trans endothelial electrical resistance (TEER) of the endothelial cell layer was measured before and after treatment. **(D)** Microscopic images of the endothelial cells from the top insert were taken before and after treatment using an EVOS® FL Imaging System. Scale bar = 200 µm. Mean ± SEM values were graphed from 3 independent experiments. **(E)** Time course penetration profile of EVG/PLGA EVG NPs across the *in vitro* BBB in presence and absence of P-gp inhibitor elacridar. **(F)** Area under the curve of EVG and PLGA-EVG penetration in presence and absence of P-gp inhibitor elacridar. Area under the curve from 0 to last time t (AUC_0–t_) was calculated using the linear trapezoidal method. Mean ± SEM values were graphed from 3 independent experiments. *Indicate p < 0.05 compared with native EVG drug in absence of P-gp inhibitor.

To understand whether nanoparticle formulation can increase the transmigration of EVG in the *in vitro* BBB model, we measured EVG amount in the bottom chamber from both media and cell lysate samples. As expected, PLGA-EVG NPs showed higher penetration compared to EVG native drug. A dose-dependent penetration profiles for both EVG native drug and PLGA-EVG NPs were observed. A significant increase of EVG penetration was observed for both free EVG and PLGA-EVG NPs from 2.5 µg/mL to 5 µg/mL and from 5 µg/mL to 10 µg/mL (Fig. 8A, *p < 0.05 compared with PLGA-Blank, #p < 0.05 compared with the previous concentration). The results showed that a maximum of 27% of PLGA-EVG NPs can cross the BBB model after 24 hours. The results also showed a significant increase in penetration of PLGA-EVG NPs compared to EVG native drug at the dose of 5 µg/mL and 10 µg/mL. We also performed a time-dependent penetration study using 5 µg/mL of native EVG/PLGA-EVG NPs. Both native drug and NPs showed a time-dependent penetration across the BBB model. Penetration of PLGA-EVG NPs across the BBB model showed a significant increase in the area under the curve (AUC) compared to EVG native drug (Fig. 8B, *p < 0.05). These results demonstrate that PLGA-EVG NPs improve the ability of EVG to cross the BBB model in the *in vitro* model. Moreover, no changes were observed for TEER values (Fig. 8C) and endothelial cell morphology (Fig. 8D) before and after the treatment, suggesting that PLGA-EVG transmigration did not change the monolayer integrity of the BBB model. Additionally, we identified the mechanistic contribution of P-gp in interfering the penetration of EVG in the *in vitro* BBB model and the capability of PLGA NPs to bypass the BBB efflux transportation. For this, we performed a penetration study in the presence and absence of a P-gp inhibitor elacridar for 24 hours (Fig. 8E). The AUC_(0-t)_ of *in vitro* BBB penetration for EVG was significantly increased in the presence of the P-gp inhibitor, suggesting that the low EVG level in the CNS is, at least in part, mediated by P-gp efflux transportation (Fig. 8F). In contrast, the penetration of PLGA-EVG NPs showed no change in the presence and absence of the P-gp inhibitor, suggesting the ability of NP to bypass the efflux transporter P-gp in the *in vitro* BBB.

### Improved viral suppression in HIV-1-infected MDM after crossing the *in vitro* BBB model

**Figure 9:**
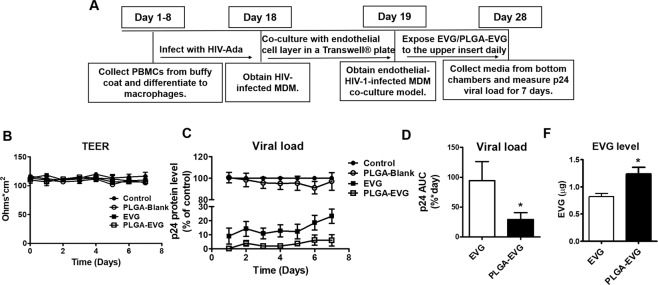
Viral suppression of EVG/PLGA-EVG in HIV-1-infected MDM after crossing *in vitro* BBB model. (**A)** Treatment paradigm for collecting HIV-1-infected MDM and performing EVG/PLGA-EVG viral suppression study. **(B)** TEER of the endothelial monolayer was measured at indicated time points. **(C)** Time course of HIV-1 replication upon drug exposure in HIV-1-infected MDM after crossing *in vitro* BBB model. All assays were performed on triplicate samples with MDM derived from two different donors. To minimize inter-donor variability, p24 levels were normalized to the control MDM, which obtained from the same donor, and reported as a percentage of the control group. **(D)** Area under the curve of EVG and PLGA-EVG. Area under the curve from 0 to last time t (AUC_0–t_) was calculated using the linear trapezoidal method. **(F)** EVG levels in MDM at the end of the treatment. EVG levels in MDM were measured from the day-7 cell lysate samples from 3 × 10^5^ cells at the end of the experiment using LC-MS/MS. Mean ± SEM values were graphed from triplicate samples with MDM derived from two different donors. *Indicate p < 0.05 compared to EVG native drug.

Since macrophages can be infected productively by HIV-1 in the CNS^33^, we examined the viral suppression efficacy of PLGA-EVG NPs in HIV-1-infected macrophages. We examined PLGA-EVG NPs and native EVG for their effects on viral replication on HIV-1-infected MDM in a modified *in vitro* BBB model following a one-month treatment paradigm as described in the treatment scheme (Fig. 9A). HIV-1-infected MDM were cultured on the bottom chamber, which will not be exposed to EVG directly. Blank PLGA NPs, EVG, and PLGA-EVG NPs were given on the upper chamber, which contains BBB monolayer. TEER values of the BBB model were measured for all the groups every day. Both EVG and PLGA-EVG NPs did not compromise the physiological integrity of the *in vitro* BBB model (Fig. 9B). HIV p24 protein level was measured from the bottom chamber daily. The effect of viral suppression in the MDM will be caused by the EVG/PLGA-EVG upon crossing the BBB monolayer to the bottom chamber. Our results showed that PLGA-Blank NPs have no effect on viral replication. However, both native EVG and PLGA-EVG NPs suppressed the viral replication to 70–100% of control (Fig. 9C). We calculated the p24 area under the curve (AUC) to compare the viral suppression efficacy of native EVG and PLGA-EVG NPs from day-1 to day-7 (Fig. 9D, *p < 0.5). The results showed that the HIV-1-infected MDM, when exposed to PLGA-EVG NPs have a significantly lower viral load, indicating that PLGA-EVG NPs have better efficacy on viral suppression in HIV-1-infected MDM after crossing the *in vitro* BBB model. We also measured EVG levels in MDM at the end of the treatment. EVG levels in MDM was significantly increased when exposed to PLGA-EVG NPs, compared with EVG native drug (Fig. 9F, *p < 0.5). This result suggests that the enhanced viral suppression efficacy, achieved by PLGA-EVG NPs, is correlated with increased EVG intracellular uptake in MDM.

## Discussion

Although HIV-1-associated dementia (HAD), the most severe form of HAND, is rare in developed countries^34^, there is no specific treatment option available for HAND^2^. Currently, ART is the best option for delaying or preventing HIV-1 disease progression^35^. However, due to the inability of ARVs to cross the BBB following systemic administration, ART is still not clinically applicable for the treatment of HAND^2^. HIV-1 infection in CNS cells affects not only cognitive functioning but also the opportunity of HIV-1 eradication^2^. Individuals with HAND commonly show characteristics of dysfunctional and impaired judgment, memory, multitasking, and attention^36^. The CNS can serve as a viral reservoir for production of HIV-1, and limit the opportunity for viral eradication^37^. Thus, there is a need for novel treatment regimens that cross the BBB and deliver therapeutic into the CNS to suppress the viral replication in the CNS cells, especially viral reservoirs. The Nair group has explored a field-controlled magnetic-NP drug formulation with the capability of magnetic field-triggered release across the BBB, for delivery of ARVs or siRNAs to the brain^38–40^. The same group has also collaborated with the El-Hage group and demonstrated an intranasal delivery of siRNA in mouse brain to achieve HIV-1 attenuation^41^. The Gendelman group also has extensive experience with ARV nanoformulation where they have developed several long-acting nanoformulated ARV prodrugs that show slow release profiles and improved antiretroviral activity^42–44^. Although these studies have demonstrated the use of nano-carriers to deliver ARVs to the brain^38–41,45^, none of these strategies are in clinic yet. Therefore, it is necessary to study and develop alternative, safe, and clinically applicable ARV delivery systems for the treatment of HAND^9,19^.

We selected EVG as our model drug, which is a FDA-approved integrase strand transfer inhibitor, and is part of the first line ART regimen for the majority of HIV-1-infected populations in the USA^46^. EVG in this nanoformulation provides an effective antiretroviral ability and a favorable safety profile. In the last three years, researchers started formulating EVG into NPs to combat HIV-1 infection^47,48^. For example, EVG has been formulated into a bioadhensive PLA NPs with a hyperbranched polyglycerols (HPG) coating on the surface^47^. These bioadhensive PLA NPs were distributed widely throughout the reproductive tract, and showed a prolonged intravaginal delivery of EVG. Moreover, EVG has been used together with tenofovir alafenamide (TFV) in a PLGA-based NPs coated with poloxamer 407 as a prevention strategy to against HIV-1^48^. This EVG NPs can extend the t_1/2_ of EVG from 10.8 h to 3.3 days and protect mice with 100% and 60% uninfected rate on day 4 and day 14 post-nanoparticle injection after a HIV-1 challenge. In contrast to the published EVG nanoparticle studies, our goal is to study the potential use of EVG NPs in CNS drug delivery to suppress the HIV-1 replication in the brain. Like other EVG NPs, we also developed a polymeric nanoparticle of EVG for a better stability and safety profile. An FDA-approved polymer, PLGA, was used to incorporate EVG and formulate the core of the NP. PLGA is a biocompatible polyester, which can be converted into water and carbon dioxide via tricarboxylic acid cycle and eventually eliminated from the body^49^. In addition, human body has shown to have low inflammatory response to PLGA suggesting its applicability for systemic administration clinically^50^. In this work, we identified that the safety and stability profile of the PLGA NPs, and both the safety and the stability profile of the PLGA NPs support their use as a potential CNS delivery strategy.

Understanding the mechanistic internalization of NPs by the targeted cells can increase our knowledge of how these particles are taken up and are transported within the cells, and to which extent they are secreted^51^. In general, most of the NPs are taken up into cells via endocytosis into endosomes and subsequently trafficked into lysosomal compartments^52^. Our data showed that a combination of different endocytosis pathways were involved in the uptake of PLGA NPs in the macrophages, which is consistent with previous studies^53–55^. Although initially present in early endosomes, PLGA NPs can still bypass the endo-lysosomal compartments in macrophages with less and no co-localization found in the presence of late endosome and lysosome marker, respectively. Our design allows the PLGA NPs escape the endo-lysosomal degradation/secretion, which helps the therapeutic to work effectively inside the cells^52^.

NPs have been demonstrated as a promising strategy to deliver drugs at the site of action, bypass transporters and drug metabolic enzymes, and cross the BBB^19,21^. Thus, we hypothesize that, EVG in PLGA NPs can bypass transporters on the BBB to perform a higher drug transmigration and can also bypass the transporters and drug metabolic enzymes on macrophages after crossing the BBB, showing improved viral suppression on the HIV-1-infected macrophages. In order to have an efficient brain delivery, we used poloxamer 188 as the surfactant in our PLGA NP formulation. Poloxamer 188 is known to have low toxicity and able to decrease inflammation in the injured brain of experimental rats^56^. Poloxamer 188 has been used as an effective surfactant for brain delivery in cancer^57,58^. Literatures showed that poloxamer 188 coating can help PLGA NPs become to an efficient brain delivery method^57^. They demonstrated that poloxamer 188-coated PLGA NPs have a high anti-tumor effect against an intracranial 101/8 glioblastoma in rats, compared with the non-coated NPs. In another study, poloxamer 188-coated PLGA NPs demonstrated the greatest cellular uptake in the brain tissues over the NPs coated with other commonly used surfactants such as polysorbate 80 and poloxamer 407^58^. Similarly, the poloxamer 188-coated EVG NPs demonstrated an effective brain delivery profile in our study.

Currently, the magnetic nanoformulation is the most commonly studied nanoformulation for NeuroAIDS^38,40^. Magnetic NPs of azidothymidine showed a 3-fold higher transmigration in an *in vitro* BBB model, compared to the native drug^38^. This magnetic nanoformulation of azidothymidine also showed a slightly lower p24 antigen production when treating with HIV-1-infected PBMCs. The same group also developed a magnetically layer-by-layer NPs with a ~37.95% BBB transmigration and ~33% p24 suppression on astrocytes *in vitro*^40^. Compared with those magnetic NPs, our PLGA-EVG NPs showed a comparable percentage of transmigration and an efficient viral suppression in HIV-1-infected macrophages. To the best of our knowledge, this is the first report of using EVG nanoformulation as a potential delivery method to cross the BBB model and suppress the HIV-1 in the CNS macrophages. We understand that the use of mouse model of the BBB with human macrophages is a limitation of this study. However, a combined model with mouse BBB cells and human macrophages fulfils the basic requirement for an *in vitro* BBB model that provides a comparison of both drug penetration and viral suppression of PLGA-EVG NPs and EVG native drugs in our study. In this model, the mouse BBB model can be used to study BBB transmigration because of the tight junction and the expression of efflux transporters P-gp and BCRP^30–32^. The validity of the BBB model in this study is further strengthen, because we are able to study the efficacy of the viral suppression in HIV-1-infected human primary macrophages^59^. Overall, compare to commonly used magnetic NPs, our PLGA-EVG NPs is easy to prepare, is FDA-approved non-toxic NPs, and does not require an application of an external magnetic field. We expect that this PLGA-EVG NPs have the potential to move forward to the clinical use.

## Conclusion

Our current study demonstrated a poloxamer-PLGA based nanocarrier is an efficient delivery approach for EVG. The PLGA-EVG NPs demonstrated a favorable stability and safety profile. The PLGA-EVG NPs demonstrated significant transmigration across an *in vitro* BBB model. Most importantly, PLGA-EVG NPs showed an improved viral suppression in HIV-1-infected macrophages after crossing the BBB model. Ongoing study in our laboratory is to determine its safety and efficacy in animal model. This is the first step towards achieving our goal to create a safe and efficient drug delivery method to target HIV reservoirs in the CNS for potential clinical.

## Material and Methods

### Materials

Active ingredient elvitegravir (EVG, E509000) was purchased from Toronto Research Chemicals Inc. (Ontario, Canada). Poly(D,L-lactide-*co*-glycolide) (PLGA) (50:50 lactide-glycolide ratio, Mw: 31,000–50,000, ester-terminated) was brought from Birmingham Polymers (Pelham, AL). The following items were obtained from Sigma-Aldrich Co. (St. Louis, MO): poloxamer 188 (pluronic F-68) (P1300, Mw: 8350), poly(vinyl alcohol) (PVA) (363138, Mw: 30,000–70,000), poly(L-lysine) (PLL) (Mw: 30,000–70,000), coumarin-6 (442631), acetone (650501), nocodazole (M1404), cytochalasin-D (Cyto-D) (C8273), chlorpromazine (CPZ) (C1240), monensin sodium salt (M5273), genistein (G6649), methyl-β-cyclodextrin (Mβ-CD) (C4555), hexadimethrine bromide (polybrene) (107689), MitoTracker^TM^ Deep Red (M22426), Texas Red^TM^ Conjugate (T2875), and CellLight^TM^ Late Endosomes-RFP (C10589). HPLC grade acetonitrile (A955) and formic acid (85178), BD PrecisionGlide 25 G needle (14–826–49) and BD 1 mL TB syringe (14-826-88) were obtained from Fisher Scientific (Hampton, NH). Sterile phosphate-buffered saline was obtained from Gibco (Dublin, Ireland). Ethylenediaminetetraacetic acid (EDTA) was brought from Boston Bio Products (Ashland, MA).

The immortalized mouse brain endothelial cells (bEnd.3, CRL-2299) and mouse astrocytes (C8-D1A, CRL-2541) were purchased from American Type Culture Collection (Manassas, VA, USA). Human monocytes-derived macrophages (MDM) were differentiated from the de-identified human blood, which was obtained from Interstate Blood Bank Inc. (Memphis, TN) upon approval from the Institutional Review Board (IRB, UTHSC). Dulbecco’s Modified Eagle’s Medium (DMEM) were obtained from American Type Culture Collection. Roswell Park Memorial Institute (RPMI) 1640 media and lymphocyte separation medium were bought from Corning Inc (Tewksbury, MA). Fetal bovine serum (FBS) was obtained from Atlanta biologicals (Atlanta, GA). L-glutamine and penicillin-streptomycin (P/S) were purchased from Fisher Scientific. The human recombinant cytokines, including M-CSF used for MDM differentiation was purchased from PeproTech (Rocky Hill, NJ), Recombinant Human IL-2 Protein was purchased from R&D system, Inc. (Minneapolis, MN). HIV-1 Ada strain was obtained from the NIH AIDS Reagent Program (Germantown, MD). RosetteSep enrichment cocktail was purchased from StemCell Technologies (Vancouver, Canada). The P24 ELISA kit (801111) was purchased from ZeptoMetrix Corp (Buffalo, NY) to assess HIV viral load in HIV-1-infected MDM.

All methods were carried out in accordance with relevant guidelines and regulations. All experimental protocols with cell lines and primary cells were approved by the Institutional Biosafety Committee (IBC) at the University of Tennessee Health Science (UTHSC). The protocols for analysis of blood samples from de-identified subjects obtained from Interstate Blood Bank (Memphis, TN) were approved by Institutional Review Board (IRB), UTHSC. The use of blood samples from de-identified subjects, bought from a blood bank, does not require informed consent from subjects and is waived from the IRB approval.

### Preparation of PLGA-EVG/PLGA-C6 NPs

EVG loaded PLGA NPs were prepared by nano-precipitation technique as described in our previous report^23^. Cells were treated with 5 µg/mL of coumarin-6 equivalent PLGA nanoparticles at different Briefly, 45 mg of PLGA was dissolved in 4 mL of acetone (organic phase). EVG (4 mg) was added to the above organic phase, under constant magnetic stirring. The PLGA-EVG organic phase was then added dropwise into 10 mL of 1% PVA-aqueous solution on a magnetic stir plate at 400 rpm. After 3 hours, aqueous solution of 10 mg of PLL and 50 mg of F-68 were added to the nanoparticle suspension and stirred at room temperature to allow acetone evaporation. Finally, the larger aggregates were removed by centrifugation at 1000 rpm for 10 min. The finer and uniform PLGA-EVG NPs were stored at 4 °C/−20 °C for further study. Coumarin-6 (C6) loaded NPs were also prepared in a similar manner as EVG loaded PLGA NPs. C6 was used as a fluorescent model to track, locate, and measure the green fluorescence for semi-quantitative and qualitative cellular uptake. We have characterized both PLGA-EVG NPs and PLGA-C6 NPs in our previous report^23^. PLGA-EVG NPs and PLGA-C6 NPs showed similar particle sizes, and PLGA-C6 NPs were used to study subcellular localization, cellular uptake and internalization, and BBB model transmigration in this study.

### *In vitro* drug release

The *in vitro* drug release study was performed using a Float-A-Lyzer® dialysis device with molecular weight cut-off of 8–10 kDa (Sigma-Aldrich Co. Z726508). A volume of 1 mL PLGA-EVG NPs formulation was enclosed in a dialysis device and incubated in 30 mL cell culture media at 37 °C under mild agitation. At 15, 30 min, and 1, 3, 6, 9, 12, 24 h, 500 µL incubation media was withdrawn and analyzed for EVG level using LC-MS/MS. The same volume of fresh media was added to the incubation media after each sampling.

### Human serum protein binding to PLGA NPs

Three hundred micrograms of PLGA NPs were incubated with 1–50% HS for 3 hours at 37 °C. The HS bound PLGA NPs were obtained by centrifugation of this NPs-HS mixed solution at 10,000 rpm for 20 min. The HS bound PLGA NPs^60^ in the pellet were denoted as % of HS@NPs. For example, the 10% HS bound PLGA NPs were recognized as 10HS@NPs. To examine the HS protein adsorption on PLGA NPs, the HS@NPs samples were either resuspended in PBS for a dynamic light scattering (DLS) analysis or lyophilized using the Labconco Freeze Dry System (−53 °C, 133 × 10^−3^ mBar; Labconco, Kansas City, MO) to perform a Fourier Transform Infrared (FTIR) spectroscopy. The HS protein adsorption on PLGA NPs was determined by sodium dodecyl sulfate polyacrylamide gel electrophoresis (SDS-PAGE) analysis as described^61^. Briefly, the adsorption capacity of HS proteins was studied based on 3 protocols: (1) equal amount (300 µg) of PLGA NPs were incubated with various concentration (1–50%) of HS for a fixed incubation period (3 h); (2) equal amount (300 µg) of PLGA NPs were incubated with a fixed concentration (10%) of HS for a various incubation period (0.5, 1, 2, 3 and 24 h); and (3) various amount (10–300 µg) of PLGA NPs were incubated with a fixed concentration (10%) of HS for a fixed incubation period (3 h). The HS@NPs were obtained by centrifugation at 10,000 rpm for 20 min. The HS@NPs samples were separated by size in a 10% SDS-PAGE analysis using the constant voltage of 150 V for 75 min. The SDS-PAGE gel was processed for a Bio-Safe™ Coomassie Stain (BioRad, Hercules, CA), and scanned using a BioRad Gel Doc.

### Dynamic light scattering

A DLS analysis was performed to measure the particle size and zeta potential of PLGA NPs or HS@NPs using a Zetasizer instrument (Nano ZS, Malvern Instruments Ltd., Worcestershire, UK). Both particle size and zeta potential of NPs/HS@NPs were measured in PBS at 25 °C. The particle size of each sample was measured for 3 min (~15 runs), and the zeta potential of each sample was measured for 90 runs. The average particle size and zeta potential of 3 readings were calculated and reported for each sample.

### Fourier transform infrared spectroscopy

FTIR spectroscopy was employed to confirm the extent of binding or presence of HS proteins on HS@NPs. The FTIR spectra of lyophilized HS@NPs were acquired using PerkinElmer Spectrum 100 FTIR spectrometer (Waltham, MA) equipped with a Diamond/ZnSe Attenuated Total Reflection crystal plate. PLGA NPs were used as a negative control, and the pure HS was used as a positive control. An average of 32 scans was performed on all the samples. The spectra were scanned from 4000 to 600 to cm^−1^ with a resolution of 4 cm^−1^.

### Stability assay

We determined the particle size and zeta potential of PLGA NPs using DLS. PLGA NPs were stored at room temperature, 4 °C, or −20 °C for a stability test. DLS analysis was performed on the room temperature sample for 7 days. The size and zeta potential of PLGA NPs from 4 °C and −20 °C were measured on day 7, and an additional measurement was performed on day 30 for the −20 °C sample. The particle size and zeta potential of PLGA NPs in 1 mM HEPES buffer from pH 1 to pH 11 were measured to investigate the alteration of PLGA NPs with this pH range.

### Hemocompatibility

The red blood cells (RBCs) were collected from the de-identified human blood. The freshly collected RBCs were resuspended in RPMI phenol red free media for a hemolysis assay as described in our previous report^61^. Briefly, the RBC suspension (600 µL) was incubated with 5–200 µg/mL EVG or equivalent concentrations of PLGA-EVG, 30HS@PLGA-EVG, and PLGA-blank, 30HS@PLGA-blank in Eppendorf tubes for 3 hours at 37 °C. After incubation, the experimental tubes were centrifuged at 1000 rpm for 10 min and the supernatant was transferred to a 96-well plate to measure the release of hemoglobin using a plate reader (Cytation 5 imaging, BioTek, Winooski, VT) at 570 nm. Ten microliter of RBCs from the pellet were imaged on a glass slide using the AMF4300 EVOS® FL Imaging System (Life Technologies, Carlsbad, CA). RBCs dissolved in MilliQ water was used as a positive control #1 (100% of hemolysis). SDS was used as positive control #2 (hemo-toxicity), and PBS was used as a negative control (0% of hemolysis) in this experiment.

### Generation of HIV-1-infected primary macrophages

Peripheral blood mononuclear cells (PBMCs) was used to generate MDM according to previously published reports^62,63^. Briefly, the buffy coats were diluted and layered over the lymphocyte separation medium and centrifuged for 20 min at 1200 g. PBMCs for MDM differentiation were collected and cultured in RPMI media containing 10% HS, 1% L-glutamine, and 5% penicillin-streptomycin. After overnight incubation, non-adherent cells were washed by PBS. The adherent PBMCs were used for the generation of macrophages. M-CSF (50 ng/mL) was added to the MDM flasks for macrophage differentiation. For HIV-infection, mature MDM were collected after 7 days of differentiation, followed by incubation with polybrene (2 µg/mL) and HIV-1 Ada strain (30 ng/1 × 10^6^ cells) for HIV-1 infection in a 12-well plate with 3 × 10^5^ cells/well. After overnight incubation, MDM were washed twice with PBS to remove the polybrene and HIV-1 Ada strain. The cells attached on the bottom of the well were considered as healthy primary MDM with initial HIV-1 infection. IL-2 (10 ng/mL) was added to the culture media of MDM to induce HIV-1 infection. After 7–10 days of the initial HIV-1 infection, cell culture media samples from MDM were collected to assess HIV-1 p24 level using a p24 ELISA kit (ZeptoMetrix Corp, Buffalo, NY) to confirm the HIV-1 infection.

### Cellular toxicity assay

Cellular toxicity assay was performed on MDM in the presence of EVG native drug, PLGA-EVG NPs, and HS30@PLGA-EVG using an XTT cell viability kit (Cell signaling, Danvers, MA). In this study, MDM were seeded into 96-well plates (2 × 10^4^ in each well). Cells were incubated with 0–20 µM of EVG or equivalent concentrations of PLGA-EVG, and HS30@PLGA-EVG for 24 hours. The cells were washed twice with PBS and replaced with 100 µL fresh media. Subsequently, 50 µL of XTT detection solution, which contains electron coupling solution, was added to each well of the 96-well plate. MDM were incubated with XTT detection solution for 3 hours and absorbance was measured using a plate reader at 450 nm. The percentage of viable cells from treatment groups was calculated by comparing the absorbance with the untreated cells. Data presented are from five replicates.

### Sub-cellular localization

In this study, PLGA-C6 NPs were used to visualize the sub-cellular localization of the NPs. MDM were seeded on 4-well cell culture chamber glass slides (CellTreat, Pepperell, MA). Cells were treated with 2.5 µg/mL of PLGA-C6 NPs for 2 hours. Subsequently, cells were incubated with markers for mitochondria (MitoTracker^TM^ Deep Red), early endosome (Texas Red^TM^ Conjugate), late endosome (CellLight^TM^ Late Endosomes-RFP), and lysosome (LysoTrackerR Red DND-99, Life Technologies) for 30 minutes. After the incubation, cells were fixed using 4% paraformaldehyde for 20 minutes and permeabilized with 0.2% TritonX-100 for 5 minutes. All the cells were stained with DAPI (Life Technologies) and mounted in Vectashield Mounting Medium (Vector Labs, Burlingame, CA) to visualize nuclei. All the images were taken using a laser confocal microscope (Carl Zeiss LSM 710, Oberkochen, Germany) under 400 × magnification using oil immersion objective.

### Cellular uptake and internalization mechanism

MDM were seeded in 6-well plates at a density of 8 × 10^5^ cells/well. The cells were pretreated with various endocytosis inhibitors for 30 min at 37 °C. The inhibitors were: nocodazole (10 µg/mL), Cyto-D (10 µg/mL), CPZ (10 µg/mL), monensin (200 nM), genistein (200 µM), and MβCD (1 mM). The cells were incubated with 2.5 µg/mL PLGA-C6 NPs for 2 hours at 37 °C. After the treatment, cells were washed twice with PBS, trypsinized, and collected to measure the cellular uptake. Semi-quantitative PLGA-C6 uptake in MDM were measured using Accuri C6 Flow Cytometer (Accuri Cytometer Inc, Ann Arbor, MI). Images were taken with a laser confocal microscope (Carl Zeiss LSM 710) under 400× magnification using oil immersion objective.

### *In vitro* BBB model

Mouse brain endothelial cells bEnd.3 and mouse astrocytes C8-D1A were cultured in complete media (DMEM media with 10% fetal bovine serum and 1% penicillin-streptomycin solution) in T75 flasks at 37 °C in a humidified incubator with 5% CO_2_ before building *in vitro* BBB model. Transwell^®^ -COL collagen-coated 0.4 μm pore polytetrafluoroethylene membrane insert (Sigma-Aldrich) was used to build an *in vitro* BBB model as previous published protocols^32,64^. Mouse astrocytes were seeded on the bottom of 12-well plates at a density of 2 × 10^5^ cells/well. After 24 hours of adhesion, mouse endothelial cells were seeded onto the upper side of the Transwell^®^- COL inserts at a density of 2 × 10^5^ cells/well, and the inserts were placed in a 12-well plate containing astrocytes. The *in vitro* BBB model was grown for 5 days to achieve ~90% confluency for transmigration assay.

### Trans endothelial electrical resistance (TEER) measurements

The integrity of the tight junction dynamics of the BBB model was measured in terms of transendothelial electrical resistance (TEER) using EVOM2 Epithelial Voltohmmeter (World Precision Instruments, Sarasota, FL)^65^. TEER values were measured regularly every day for 6 days from the day of cell seeding. The TEER values were also measured during the experiment period to assess the integrity of the endothelial cell layer. TEER of the blank insert with media alone was subtracted from the TEER final reading to obtain the TEER value for the endothelial monolayer. The TEER value was presented as the value of resistance (current, Ohm) multiplied by the area (cm^2^) of the endothelial monolayer (Ohm⋅cm^2^).

### Transmigration study of PLGA NPs

The transmigration of PLGA NPs was visualized and measured using fluorescence dye C6 in a confluent *in vitro* BBB model. PLGA-C6 NPs at concentrations of 2.5, 5, and 10 µg/mL were added to the upper inserts and incubated at 37 °C for 24 hours. After incubation, astrocytes in the bottom chamber were washed twice with PBS. The media of the bottom chamber was measured using a fluorescence plate reader (SpectraMAX M2e; Molecular Devices, San Jose, CA) with excitation at 488 nm and emission between 400 and 650 nm. The transmigration of PLGA-C6 NPs was presented in terms of fluorescence intensity. C6 concentration in the media was also calculated using a C6 calibration curve with the matrix of media. The images of receiving astrocytes were taken by EVOS® FL Imaging System.

### Penetration of PLGA-EVG NPs in the *in vitro* BBB model

The *in vitro* BBB model was used to assess the penetration of the PLGA-EVG NPs. We performed a 24-hour EVG native drug/PLGA-EVG NPs treatment on the BBB cells. EVG native drug/PLGA-EVG NPs at concentrations of 2.5, 5, and 10 µg/mL were added to the upper inserts and incubated at 37 °C for 24 hours. A sample of 50 µL of receiver media was collected at 1, 3, 6, 9, and 24-hour time point, and an equal volume of fresh media was added. At the end of the experiment, the cells from the bottom chambers were collected in RIPA buffer as the cell lysate. The amount of EVG flux to the bottom chambers from both media and cell lysate was measured using our developed LC/MS method^66^.

### Quantification of EVG using LC-MS/MS

A 50 µL of media or cell lysate samples were mixed with 3-volumes of cold acetonitrile containing internal standard (IS) ritonavir (50 ng/mL). The mixed solution was vortexed and centrifuged at 8000 rpm for 10 min for protein precipitation. A clear supernatant was collected and analyzed using our established LC-MS/MS method^66^. Briefly, a Shimadzu liquid chromatographic system (Kyoto, Japan) coupled with an AB SCIEX Triple Quad 5500 tandem mass spectrometer (Framingham, MA) was used for the analysis. Chromatographic separation was performed on an Xterra® MS C18 column (125 Å, 3.5 μm, 4.6 mm × 50 mm; Waters, Milford, MA). The mobile phase used for EVG separation consisted of (A) water with 0.1% formic acid and (B) acetonitrile with 0.1% formic acid (v/v) at a flow rate of 1 mL/min. The gradient elution was as follows: 0–1.5 min, 50% B; 1.5–5.1 min, 60% B (v/v). The quantification of the validated assay of EVG was 1 to 500 ng/mL. EVG and IS were eluted separately at approximately 3.27 and 2.72 min, respectively. The multiple reactions monitoring (MRM) transitions (m/z) Q1/Q3 selected for quantitative analyses were 447.9/343.8 for EVG and 721.3/296.1 for the IS. In order to reduce matrix effects, calibration curves were prepared with blank media or cell lysate based on the sample types.

### Mechanistic contribution of P-gp in interfering the penetration of EVG in the *in vitro* BBB model

The mechanistic contribution of P-gp in interfering with the penetration of EVG was assessed in the presence and absence of the P-gp inhibitor elacridar. The *in vitro* BBB layer was preincubated with/without elacridar (500 nM) for 30 min before the penetration study. After preincubation, 5 µg/mL of EVG native drug/PLGA EVG NPs were added to the upper inserts and incubated at 37 °C for 24 hours. A sample of 100 µL of media from the bottom chamber was collected at 1, 3, 6, 9, and 24-hour time point, and an equal volume of fresh media was added. The amount of EVG flux to the bottom chambers was measured using LC-MS/MS method.

### Viral suppression of PLGA-EVG NPs in HIV-1-infected MDM after crossing *in vitro* BBB model

To determine the efficacy of the viral suppression of PLGA-EVG NPs, we used HIV-1-infected primary MDM to create a modified *in vitro* BBB model in a Transwell^®^ plate. The confluent brain endothelial monolayer was obtained as described in the previous section. The upper insert of the *in vitro* BBB model containing confluent brain endothelial cells was transferred to the 12-well plate containing HIV-1 infected MDM, and co-cultured with HIV-1-infected MDM. In this modified *in vitro* BBB model, HIV-1-infected MDM were cultured on the bottom of the 12-well plate. Endothelial monolayers in the upper inserts were exposed to control, blank PLGA NPs, EVG native drug (5 µg/mL), and PLGA-EVG NPs (5 µg/mL) for 7 days. HIV-1 viral loads were measured every day using a p24 ELISA kit from the culture media from the bottom chamber. The upper inserts were replaced with fresh BBB cells after 3 days to keep BBB monolayer confluent. A mean TEER value of 100 to 120 Ohms/cm^2^ that was obtained every day, was consistent with the modified BBB model during the time of the experiment. EVG levels in MDM were measured from the day-7 cell lysate samples at the end of the experiment using LC-MS/MS. All the HIV-1 related experiments were performed in the BSL-3 laboratory in the Regional Biocontainment Laboratory (RBL) of UTHSC.

### Statistical analysis

All graphs and statistical analyses were performed using GraphPad Prism 5 (GraphPad Software; La Jolla, CA). Student’s t-test analysis was used for comparisons between two groups. The statistical significance among treatments was determined from one-way ANOVA post-hoc Tukey HSD Test. A p-value ≤ 0.05 was considered significant.
